# Giant Panda Feces-Derived *Weissella confusa* BSP201703 Protects Mice Against Chronic ETEC Infection by Repairing Intestinal Barrier Function

**DOI:** 10.3390/vetsci13040382

**Published:** 2026-04-15

**Authors:** Yan Zeng, Lvchen Xiong, Yi Zhou, Jie Wang, Lei Liu, Ziyao Zhou, Bo Jing, Kangcheng Pan, Dong Zeng, Zhijun Zhong, Xueqin Ni

**Affiliations:** 1 Animal Microecology Institute, College of Veterinary, Sichuan Agricultural University, Chengdu 611130, China; yanzeng@sicau.edu.cn (Y.Z.); 18227551975@163.com (L.X.); zhouyisic@163.com (Y.Z.); w1468352947@foxmail.com (J.W.); liulei_30@foxmail.com (L.L.); jingbooo@163.com (B.J.); pankangcheng71@126.com (K.P.); zend@sicau.edu.cn (D.Z.); 2 Key Laboratory of Animal Disease and Human Health of Sichuan, College of Veterinary Medicine, Sichuan Agricultural University, Chengdu 611130, China; zzhou@sicau.edu.cn

**Keywords:** *Weissella confusa*, chronic ETEC infection, giant panda, intestinal mucosal repair, intestinal barrier function

## Abstract

Enterotoxigenic *Escherichia coli* (ETEC) causes severe intestinal infections in animals and threatens public health under the One Health framework. Most conventional studies focus on acute short-term ETEC infection, while natural persistent colonization oftern induces chronic intestinal mucosal compensatory remodeling in hosts. This study evaluated the protective effects of giant panda-derived *Weissella confusa* BSP201703 against chronic ETEC-induced intestinal damage using a giant panda fecal microbiota-associated (GPF) mouse model. Seventy-two Kunming mice were divided into six groups: blank control (C1), GPF control (C2), ETEC control (C3), and three *W. confusa* BSP201703 groups at low (1.0 × 10^7^ cfu/mL, W1), medium (1.0 × 10^8^ cfu/mL, W2), and high (1.0 × 10^9^ cfu/mL, W3) doses. Mice were first subjected to continuous ETEC challenge for 5 days to establish stable chronic intestinal injury, followed by a subsequent 5-day intervention with probiotic or sterile PBS for repairing existing damage. Growth performance, histopathology, serum D-lactate, SIgA, tight junction genes (ZO-1, Occludin, Claudin-1), and gut microbiota were analyzed. Histomorphologically, the chronic ETEC challenge induced compensatory increases in ileal villus height and crypt depth, which differed from typical acute necrotic atrophy. *W. confusa* BSP201703 mitigated ETEC-induced damage, reduced serum D-lactate (*p* < 0.05), increased SIgA, and upregulated tight junctions (*p* < 0.05). Microbial results demonstrated that medium-dose W2 maximized microbial diversity, while W1/W3 selectively enriched beneficial Bacteroidetes, *Clostridium* cluster IV, and *Clostridium* cluster XIVa taxa, confirming that moderate doses yielded optimal protection. In conclusion, *W. confusa* BSP201703 relieves ETEC injury by enhancing intestinal barrier function and regulating gut microbiota, highlighting its potential as a wildlife probiotic for One Health applications.

## Simple Summary

This study established a giant panda fecal microbiota-associated mouse model with chronic *Escherichia coli* (ETEC) infection to evaluate the repairing effect of the native strain *Weissella confusa* BSP201703 against persistent intestinal mucosal damage. Unlike acute infection, chronic ETEC challenge induced compensatory intestinal structural changes in mice. This probiotic strain alleviated established chronic intestinal lesions and restored impaired intestinal barrier function, with optimal efficacy observed at low and medium doses. These findings support the application of wildlife-derived probiotics for the rehabilitation of persistent intestinal diseases, further complementing the One Health strategy.

## 1. Introduction

As a globally vulnerable species, the giant panda (*Ailuropoda melanoleuca*) feeds on bamboo containing cellulose, hemicellulose, and pectin [1,2], but retains carnivorous digestive traits [3]. This mismatch between their diet and digestive physiology renders giant pandas susceptible to intestinal diseases, with risk increasing during dietary shifts [4,5]. Pathogenic microorganisms and drug-resistant bacteria, such as *Escherichia coli* and *Salmonella*, are responsible for the high incidence and intractable nature of intestinal disorders in giant pandas, domestic pets, livestock, poultry, and other wildlife [6,7,8,9]. Among these pathogens, enterotoxigenic *E. coli* (ETEC) and enterohemorrhagic *E. coli* (EHEC) induce intestinal mucosal damage, reduce beneficial microbiota abundance, and impair host immune function. Persistent pathogenic infection exacerbates host illness, may lead to death, and promotes drug resistance in pathogens. Drug-resistant strains such as extraintestinal pathogenic *E. coli* induce gastrointestinal diseases, disrupt intestinal microecological balance, facilitate the horizontal transfer of antibiotic resistance genes, and result in clinical antibiotic therapy failure. As a diarrhea pathogen, ETEC causes pathological damage to the jejunal and ileal mucosa, with lesions in the ileum [10]. Previous studies have isolated ETEC strains of sequence type 48 (ST48) harboring the adhesin gene papA from giant panda feces, confirming ETEC as a main pathogen associated with diarrhea in giant pandas [8,11,12]. These issues threaten the health of the giant panda population and contradict the globally advocated “One Health” concept.

Most basic ETEC-related studies focus on short-term acute challenge models within 24 to 72 h and mainly reflect immediate intestinal damage features. Captive and wild giant pandas usually carry long-term ETEC colonization and develop persistent chronic infection. Their intestinal mucosal pathological changes differ greatly from those caused by acute injury and can present special phenotypes such as compensatory intestinal epithelial hyperplasia.

Currently, probiotics are defined as live microorganisms that confer health benefits to the host when administered in adequate amounts and serve as green strategies for preventing and controlling intestinal disorders [13,14]. Their core protective mechanisms include maintaining intestinal microbial homeostasis, inhibiting pathogenic bacteria’s adhesion and colonization, regulating inflammatory responses, and preserving intestinal barrier integrity. Host-derived probiotics show colonization capacity and probiotic efficacy [15], making the development of autochthonous probiotics important for the disease prevention and control of rare wild animals like the giant panda. Due to the rarity of these species and ethical constraints, clinical trials cannot be conducted directly. Previous transcriptomic studies on blood monocytes have demonstrated conservation of Toll-like receptors (TLRs) in giant pandas and mice [16], laying a theoretical foundation for probiotic research on giant pandas using mouse models. Mouse models, including colitis models and fecal microbiota transplantation models, have become tools for studying intestinal probiotics and host health in such wildlife [17,18].

Germ-free (GF) mice serve as the gold standard for gut microbiota transplantation research, but their cost restricts widespread application. Pseudo-germ-free mice can be generated via sequential antibiotic administration, and their ability to acquire exogenous gut microbiota and recapitulate host-specific phenotypes has been well documented [19,20,21]. In the present study, a giant panda fecal microbiota-associated (GPF) mouse model was established using antibiotic-pretreated pseudo-germ-free mice to mimic the intestinal microenvironment of giant pandas, enabling efficacy evaluation in a relevant context.

The intestinal barrier consists of mechanical, immune, and microbial compartments. It plays an essential role in maintaining health for both humans and animals. Tight junction proteins such as ZO-1, Occludin, and Claudin-1 form the core structure of the mechanical barrier [22]. These proteins seal gaps between intestinal epithelial cells. Secretory immunoglobulin A (SIgA) acts as a key part of mucosal immune defense. It is indispensable for intestinal immune protection [23]. Stable gut microbiota closely support normal barrier function. Regulating microbial balance serves as one major way for probiotics to exert beneficial effects. Damage to the intestinal barrier often leads to gastrointestinal diseases. Restoring an intact barrier structure is critical for host recovery. This finding has been widely confirmed in previous studies.

Research on *Weissella confusa* remains limited, especially regarding its ability to control ETEC and protect intestinal health in rare wild animals. In vitro studies show that this strain can inhibit pathogens, produce polysaccharides, and tolerate low-acid environments [24,25]. Its functional properties are comparable to those of commonly used commercial probiotics. Previous studies indicated that human-related *Weissella cibaria* can relieve intestinal epithelial damage induced by LPS [26,27]. It also strengthens intestinal mucosal barrier performance. However, few studies focus on the barrier protective effects of giant panda-derived *Weissella confuse*. No relevant evidence explains its repairing potential against intestinal injury caused by chronic *E. coli* infection. The strain *W. confuse* BSP201703 (CCTCC NO:M2018553), used in this study, was isolated from healthy giant panda feces. Its basic probiotic characteristics have already been verified in vitro [24].

In vivo studies on wild animal-sourced probiotics are still insufficient. This study explored the functional effects of the selected strain. A giant panda fecal microbiota-associated GPF mouse model was established to simulate the natural intestinal microenvironment of giant pandas. The protective and repairing effects of *W. confuse* BSP201703 against chronic ETEC infection were evaluated in mice. Related mechanisms were further analyzed, mainly focusing on intestinal barrier improvement and gut microbiota regulation. The findings provide reliable experimental support for applying wild animal-derived probiotics in veterinary practice. It also offers valuable references for preventing intestinal infectious diseases in wild animals and livestock under the One Health framework.

## 2. Materials and Methods

### 2.1. Bacteria Strains and Preparation

*W. confusa* BSP201703 (CCTCC NO:M2018553) was cultured in De Man, Rogosa, and Sharp (MRS) broth at 37 °C for 24 h. Enterotoxigenic *Escherichia coli* (ETEC) CVCC196, provided by the China Institute of Veterinary Drug Control (Beijing, China), was cultured in Luria–Bertani (LB) broth supplemented with 5% fetal bovine serum (FBS) at 37 °C for 12 h. Both strains were harvested by centrifugation at 6000× *g* for 10 min at 4 °C, washed twice with sterile phosphate-buffered saline (PBS), resuspended in the same buffer, and adjusted to final concentrations of 1.0 × 10^7^, 1.0 × 10^8^, and 1.0 × 10^9^ cfu/mL (*W. confusa* BSP201703 for oral administration) and 1.0 × 10^9^ cfu/mL (ETEC CVCC196 for infection challenge), respectively.

### 2.2. Animal and Experimental Design

A total of seventy-two 3-week-old male Kunming mice weighing 15 ± 1.0 g were purchased from Chengdu Dashuo Biological Institute (Chengdu, China). Prior to the experiment, all mice were acclimated for 7 days with free access to standard chow and sterile water to minimize stress responses. The mice were housed in a specific pathogen-free (SPF) environment with a controlled temperature of 22 ± 2 °C and humidity of 55 ± 5%, under a 12 h light/dark cycle. The study protocol was approved by the Laboratory Animal Welfare and Ethics Committee of Sichuan Agricultural University, which holds the laboratory animal use license (SYXK (chuan) 2014-187). The official ethical approval number is 20170521. All efforts were made to minimize animal suffering and maintain the highest standards of animal welfare throughout the experiment.

**Figure 1:**
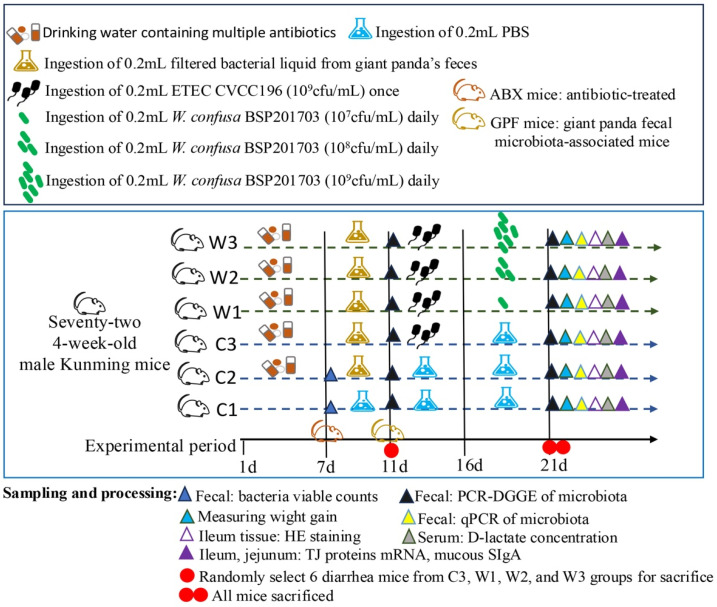
Experimental design.

Mice in group C1 received normal drinking water. For the other groups, mice were administered a mixture of antibiotics (ampicillin 200 mg/L, streptomycin 200 mg/L, neomycin 200 mg/mL, vancomycin 100 mg/L) via drinking water for 7 days to deplete the indigenous gut flora (Figure 1). Feces from groups C1 and C2 were then collected for viable cell counting of total anaerobic bacteria, total aerobic bacteria, Enterobacteriaceae, Enterococcus, *Lactobacillus*, and *Bifidobacterium*. Previous studies have shown that such antibiotic treatment reduces overall bacterial abundance and significantly decreases the abundance of specific taxa relevant to this study, such as Enterobacteriaceae associated with *E. coli* [28,29]. This approach therefore allows a comprehensive evaluation of whether the antibiotic-treated mouse model (ABX mice) was successfully established.

Subsequently, the mice receiving antibiotic treatment were gavaged with fecal suspension from healthy giant pandas (0.2 mL per mouse daily) for 4 consecutive days to establish a mouse model of giant panda fecal microbiota transplantation (GPF mice). The Mice in group C1 were administered 0.2 mL of PBS solution by intragastric gavage. Fecal samples were collected from groups C1, W1, and healthy giant pandas for PCR-DGGE analysis to evaluate the similarity of the microbial communities. The successful establishment of the giant panda fecal microbiota-associated mice model (GPF mice) was comprehensively verified based on a microbial similarity exceeding 49% and the clearance of Enterobacteriaceae in the antibiotic-pretreated mice [29,30,31].

A measure of 0.2 mL of ETEC CVCC196 suspension was administered intragastrically once on the first day to mice in groups C3, W1, W2, and W3. All mice were then raised for 5 consecutive days to develop persistent chronic intestinal injury. Diarrhea symptoms and related sample information were recorded during this period. Meanwhile, mice in groups C1 and C2 received 0.2 mL sterile phosphate-buffered saline (PBS) by gavage daily for 5 days. After the persistent injury model was established following the single ETEC challenge, mice in W1, W2, and W3 were given 0.2 mL of *W. confusa* BSP201703 suspension by daily oral gavage for the subsequent intervention period. The body weight of each mouse was recorded on two days throughout the whole experiment.

### 2.3. Sample Collection

After fecal sample collection, all mice were euthanized by cervical dislocation. Blood samples were collected via eyeball enucleation immediately afterwards. Jejunum and ileum tissues from each group were freshly harvested. Segments (1 cm in length) of the jejunum and ileum were fixed in 4% paraformaldehyde for subsequent experiments. The remaining intestinal tissues were snap-frozen in liquid nitrogen. These liquid nitrogen-frozen samples were then transferred to a −80 °C refrigerator for long-term storage.

### 2.4. Intestinal Flora Viable Count

On day 7 of antibiotic treatment, 1 g of fecal samples from the control group and the antibiotic-treated group was weighed. Each sample was resuspended in 1 mL of sterile PBS (pH 7.4) and thoroughly homogenized. Selective culture media for Enterococcus, Enterobacteriaceae, *Lactobacillus*, *Bifidobacterium*, total anaerobic bacteria, and total aerobic bacteria were used for viable bacterial counting. A 100 μL aliquot of each fecal homogenate was inoculated onto the corresponding selective medium. The inoculated plates were incubated at 37 °C for 24 h. After incubation, the colonies of each bacterial genus were counted and recorded.

### 2.5. Serum D-Lactate and Mucosal SIgA Concentration Determination

Mouse serum samples were prepared by centrifuging collected blood at 1000× *g* for 5 min at 4 °C. The concentrations of serum D-lactate and mucosal secretory immunoglobulin A (SIgA) in the jejunum and ileum were determined using commercial ELISA kits (Omega Bio-tek, Inc., Norcross, GA, USA). All experimental procedures were performed strictly in accordance with the manufacturer’s instructions.

### 2.6. Relative Quantitative Real-Time PCR (qPCR) for Tight Junction (TJ)Genes

Total RNA was extracted from liquid nitrogen-frozen jejunum and ileum tissues using RNAiso Plus (TaKaRa, Dalian, China), strictly following the manufacturer’s instructions. RNA integrity was assessed via 1% agarose gel electrophoresis. RNA purity was determined using a NanoDrop spectrophotometer (NanoDrop Technologies, Wilmington, DE, USA). Then, 1 μg of total RNA was reverse-transcribed into cDNA using the PrimeScript^®^ RT Reagent Kit with gDNA Eraser (TaKaRa, China) in accordance with the manufacturer’s protocol. The obtained cDNA was stored at −80 °C prior to qPCR analysis. The β-actin gene was selected as the internal reference gene for qPCR normalization. All primers used in this study are listed in Table 1.

Quantitative real-time PCR was performed on a CFX Connect™ Real-time PCR Detection System (Bio-Rad, Hercules, CA, USA) using the SYBR Premix Ex Taq™ II PCR kit (TaKaRa, China). Each qPCR reaction was prepared in a total volume of 20 μL, containing 10 μL of 2× SYBR Premix Ex Taq™ II, 0.4 μL of forward primer (10 μM), 0.4 μL of reverse primer (10 μM), 2 μL of template cDNA (50 ng/μL), and 7.2 μL of RNase-free water. The final concentration of each primer in the reaction mixture was 0.2 μM. The PCR thermal profile was set as follows: initial denaturation at 95 °C for 1 min, followed by 40 cycles of denaturation at 95 °C for 15 s, annealing at 60 °C for 30 s, and extension at 72 °C for 30 s. Melting curve analysis was performed after amplification to confirm the specificity of the PCR products. All reactions were run in triplicate. Relative gene expression levels were calculated using the 2−ΔΔCt method. The ΔΔCt value was defined as follows: ΔΔCt = (Ct target-Ct, β-actin) treatment group—(Ct target-Ct, β-actin) control group.

### 2.7. Histopathological Analysis

Ileum tissue segments fixed in 4% paraformaldehyde were embedded in paraffin. The paraffin blocks were sectioned and stained with hematoxylin and eosin (H&E). Histopathological changes in the ileum were observed under a light microscope. Villus length and crypt depth of the ileum were measured using image analysis software calibrated with a microscopic scale. For each sample, at least 10 non-overlapping, well-oriented visual fields were selected for measurement. All morphological parameters were expressed in micrometers (μm).

### 2.8. PCR-DGGE Analysis

Total DNA was extracted from fecal samples using a stool DNA extraction kit (OMEGA) according to the manufacturer’s instructions. The V3 region of the fecal microbiota 16S rRNA gene was amplified by PCR using specific primers. Following PCR amplification, denaturing gradient gel electrophoresis (DGGE) was performed using an electrophoresis system (Bio-Rad, Hercules, CA, USA). Polyacrylamide gels containing a 35–65% formamide denaturing gradient were prepared for separation. Electrophoresis was initiated at 200 V for 5 min, followed by a constant run at 100 V and 60 °C for 16 h. Upon completion of electrophoresis, the gels were stained using silver staining. Gel imaging was performed using a Gel Doc XR Imaging system (Bio-Rad, Hercules, CA, USA) to capture the DGGE profiles.

### 2.9. Quantitative Real-Time PCR (qPCR) Detection of Intestinal Flora

Fecal DNA was extracted from mice using an OMEGA kit, and its concentration and purity were determined using a NanoDrop^®^ ND-1000 spectrophotometer. The specificity and melting temperature (Tm) of all primers (Table 2) were verified. Quantitative real-time PCR was performed in a 20 μL reaction mixture containing 2 μL of mouse fecal DNA, 0.4 μL of forward primer (10 μM), 0.4 μL of reverse primer (10 μM), 10 μL of 2× SYBR Premix Ex Taq™ II, and 7.2 μL of ddH_2_O, with a no-template negative control included. The PCR program was set as follows: pre-denaturation at 95 °C for 1 min; 40 cycles of denaturation at 95 °C for 15 s, annealing at 55–95 °C for 30 s, and extension at 72 °C for 30 s; followed by a final extension at 72 °C for 5 min. A standard curve was established using serially diluted mouse fecal DNA templates at the determined optimal Tm value. Real-time fluorescence quantitative detection was conducted on a CFX Connect™ Real-Time PCR Detection System (Bio-Rad, Hercules, CA, USA) with three technical replicates per sample. The copy number of bacterial DNA in fecal samples was calculated based on the corresponding Ct values and the standard curve. The bacterial abundance was expressed as the logarithm of bacterial DNA copy number per gram of fecal content.

### 2.10. Statistical Analysis

Statistical analysis and plotting were performed using GraphPad Prism 10.3.0 software. The results were presented in the form of mean ± standard deviation (x ± s). Comparisons of significant differences between groups were conducted using one-way and two-way analysis of variance, with post hoc pairwise comparisons performed via Tukey’s multiple comparisons test and Šídák’s multiple comparisons test, separately. Statistical significance was defined as <0.05 or when the corresponding 95% confidence intervals (95% CIs) for mean differences did not include zero. *p* values between 0.01 and 0.05 were marked with “*”; those between 0.001 and 0.01 with “**”; those between 0.0001 and 0.001 with “***”; and *p* values < 0.0001 were labeled “****” to indicate extreme significance. *p*-values ≥ 0.05 indicated no significant difference and were not presented in the figures. PCR-DGGE profiles were analyzed using Quantity One 4.6.9 software (Bio-Rad, Hercules, CA, USA).

## 3. Results

### 3.1. Establishment and Validation of the GPF Mouse Model

**Figure 2:**
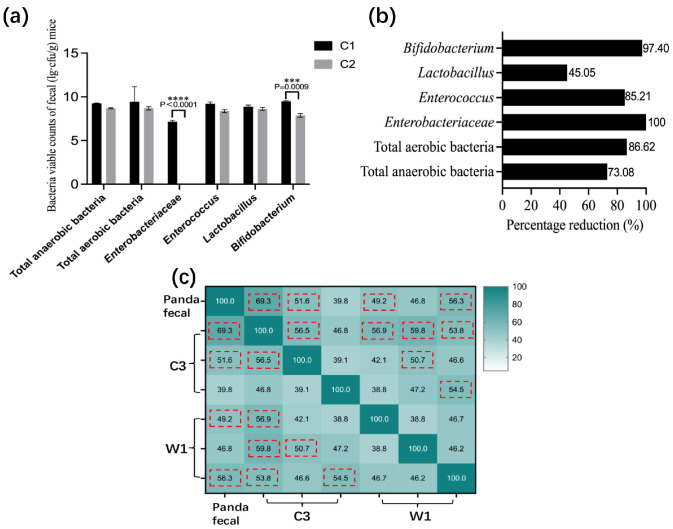
Antibiotic effects on mouse fecal bacteria and gut microbiota similarity after giant panda FMT (PCR-DGGE). (**a**) Viable counts of Enterococcus, Enterobacteriaceae, Lactobacillus, Bifidobacterium, total anaerobic bacteria, and total aerobic bacteria in feces of C1 and C2 mice (C2: antibiotic-treated, ABX mice). (**b**) Percentage reduction in viable bacterial counts. (**c**) Gut microbiota similarity (PCR-DGGE) between giant panda feces and groups C3/W1 to validate the GPF mouse model. Similarity between lanes was calculated using the Dice coefficient. The red dashed box indicates similarity > 49% (literature criterion). Combined with 100% Enterobacteriaceae clearance in C2 after antibiotics, model success was assessed.

Fecal plate counting revealed that antibiotic-treated C2 mice exhibited markedly reduced bacterial loads (45–100% clearance) compared with untreated C1 mice, especially for Enterobacteriaceae (*p* < 0.0001) and *Bifidobacterium* (*p* = 0.0009) (Figure 2a). Enterobacteriaceae were eliminated in group C2 (100% clearance; Figure 2b), confirming successful microbiota depletion. PCR-DGGE analysis showed that the gut microbiota similarity between giant panda feces and recipient mice exceeded 49% (Figure 2c). Based on these criteria, the giant panda fecal microbiota-associated (GPF) mouse model was successfully established.

### 3.2. W. confusa BSP201703 Mitigates ETEC-Induced Growth Retardation and Ileal Injury in Mice

**Figure 3:**
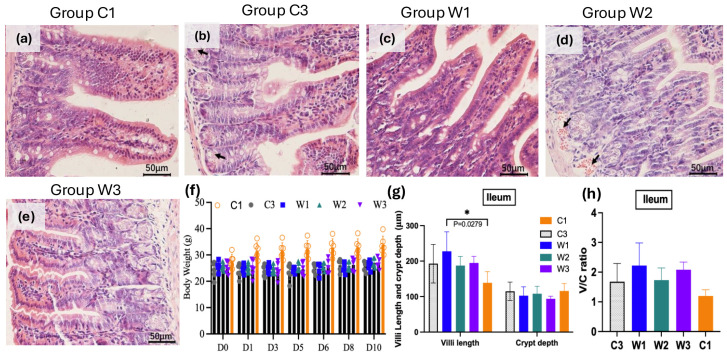
Mouse body weight and ileal H&E staining results. (**a**–**e**) Hematoxylin and eosin (H&E) staining of ileum C1, C3, W1, W2, and W3 mice (×200 magnification), the black arrow indicates slight bleeding in the submucosal layer. (**f**) Body weight gain curve of mice in each group (*n* = 6). (**g**) Ileal villi length, crypt depth, and (**h**) their ratio in mice (mean ± SD, *n* = 3).

The ileum is the main target tissue damaged by ETEC. In this study, hematoxylin and eosin (H&E) staining was performed on ileal tissues from each group (Figure 3a–e). No mortality occurred during the whole experiment. The blank control group (C1) maintained the best overall growth status. After a single initial ETEC challenge followed by prolonged chronic colonization, mice in the W2 and W3 groups gained obvious body weight within 10 days with probiotic intervention (Figure 3f). Histopathological observations revealed mild mucosal hemorrhage in the C3 model group. No obvious pathological damage was found in C1 and the three probiotic groups (Figure 3a–e). This result indicated that *W. confusa* BSP201703 could effectively relieve growth retardation caused by persistent ETEC infection.

Compared with the blank control group C1, all ETEC-challenged groups showed higher ileal villus height and villus height/crypt depth ratio (Figure 3g,h). This change reflected the typical compensatory epithelial hyperplasia formed during prolonged chronic infection rather than acute necrotic atrophy. Supplementation with *W. confusa* BSP201703 continuously optimized intestinal morphological structure and further increased villus height, crypt depth, and their ratio in W1 and W3 groups. The W1 group in particular presented significantly higher villus height compared with C1 (*p* = 0.0279). In conclusion, *W. confusa* BSP201703 can improve the abnormal ileal mucosal morphology induced by chronic ETEC infection. Some indicators reached statistical significance while others showed clear protective and reparative trends. These findings further confirm that the strain can repair intestinal mucosal remodeling caused by long-term ETEC infection.

### 3.3. Serum D-Lactate Concentration in Mice

**Figure 4:**
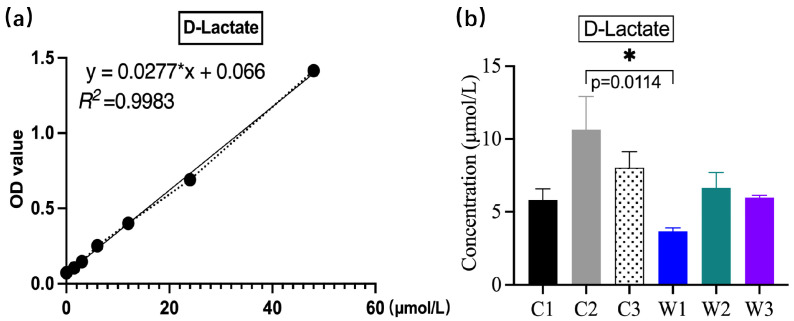
Serum D-lactic acid detection in mice by ELISA. (**a**) Standard curve of D-lactic acid. (**b**) Bar chart of serum D-lactic acid concentration in mice of each group.

Serum D-lactate was measured using an ELISA kit (Figure 4). The D-lactate concentration was significantly highest in group C2 (*p* < 0.05) and moderate in group C3. Antibiotic treatment alone severely impaired intestinal barrier integrity and increased permeability, thereby elevating circulating D-lactate levels, while subsequent pathogenic challenge induced diarrhea and accelerated its clearance. Notably, only the W1 group showed a significantly lower D-lactate concentration than group C2 (*p* = 0.0114). These findings indicate that the probiotic alleviates intestinal mucosal injury, regulates gut microbiota, reduces inflammation, and consequently decreases the production and accumulation of D-lactic acid. All three probiotic-treated groups (W1–W3) had lower D-lactate levels than group C3, although these differences were not statistically significant (*p* > 0.05). Given that serum D-lactate is positively correlated with intestinal permeability, these results suggest that low-dose *W. confusa* BSP201703 effectively improves intestinal barrier function.

### 3.4. Mucosal SIgA Concentrations in the Jejunum and Ileum

**Figure 5:**
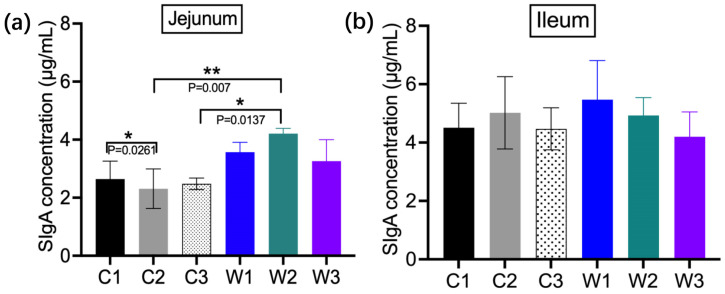
ELISA detection of SIgA in mouse jejunal (**a**) and ileal (**b**) mucosa.

Ileal intestinal secretory immunoglobulin A (SIgA) concentrations were generally higher than those in the jejunum (Figure 5). All *W. confusa* BSP201703-treated groups showed higher SIgA levels than the control groups, with the W1 group showing the highest concentration in the ileum. In the jejunum, the W2 group had the highest SIgA content, which was significantly higher than that in the C2 group (*p* = 0.007). A significant difference was also observed between the C1 and C2 groups (*p* = 0.0261). These results demonstrate that *W. confusa* BSP201703 enhances mucosal immune function and improves resistance to ETEC infection.

### 3.5. mRNA Expression Levels of Tight-Junction (TJ)-Related Proteins

**Figure 6:**
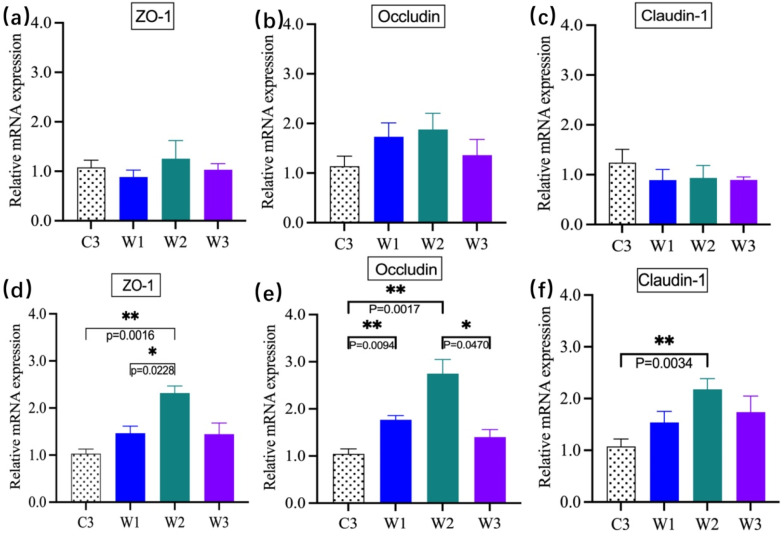
mRNA expression levels of tight junction proteins in the jejunum (**a**–**c**) and ileum (**d**–**f**) of mice. ZO-1 (Zonula Occludens-1), Occludin, and Claudin-1 are core components of intestinal epithelial tight junctions (TJ).

Compared with C3, *W. confusa* BSP201703 upregulated mRNA expression of ZO-1, Occludin, and Claudin-1 in the jejunum and ileum, with significant differences observed in the ileum (*p* < 0.05) (Figure 6). Occludin expression in the jejunum was significantly increased in W2 compared with C3. These results indicate that *W. confusa BSP201703* enhances the intestinal mechanical barrier by upregulating the expression of tight junction-related genes, with significant effects observed in the ileum.

### 3.6. Fecal Microbiota Diversity and Abundance

**Figure 7:**
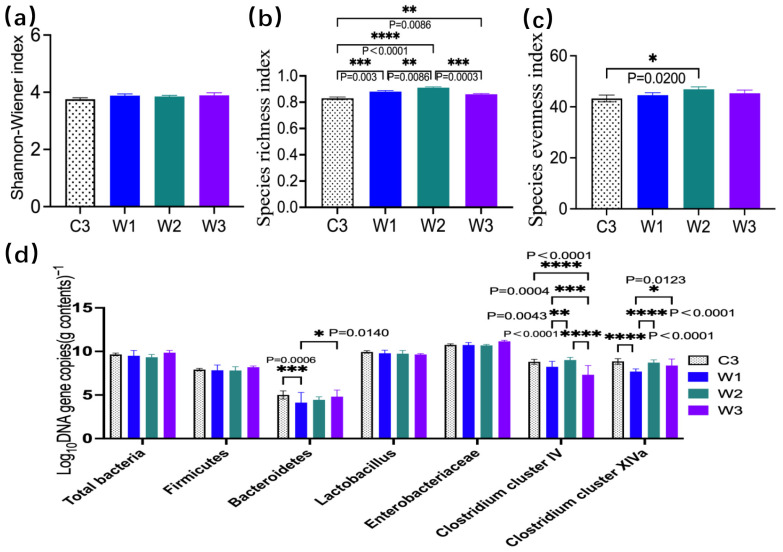
Mouse fecal microbiota diversity and the abundance of six bacterial taxa. (**a**–**c**) PCR-DGGE analysis of the Shannon–Wiener index, species richness, and evenness index. (**d**) qPCR analysis of the fecal microbiota.

The α-diversity results of intestinal microbiota in four groups of ETEC infection models analyzed by PCR-DGGE showed that the Shannon diversity, species richness, and evenness in the three *W. confusa* BSP201703 groups presented an obvious upward trend compared with the C3 group, suggesting that this probiotic could significantly improve intestinal species richness (*p* < 0.05, Figure 7a–c). The W2 group exhibited the best repairing effect on the abundance of microbiota, with a significant difference from the C3 group (*p* < 0.05). Comparison results of *Lactobacillus* and Enterobacteriaceae indicated that the abundances of the above two microbiota fluctuated gently among the four groups, with no significant inter-group differences (*p* > 0.05). Further qPCR quantitative detection of six core functional intestinal microbiota (Figure 7d) demonstrated that the probiotic could effectively promote the enrichment of intestinal beneficial bacteria, and significantly upregulate the abundances of key homeostatic microbiota in the host intestine, such as Bacteroidetes, *Clostridium* cluster IV, and *Clostridium* cluster XIVa (*p* < 0.05). The abundance of Bacteroidetes was significantly upregulated in the W1 group (*p* = 0.006) and the W3 group (*p* = 0.0140). Although the W2 group slightly increased the abundances of the two *Clostridium* clusters, no significant difference was observed compared with the C3 group (*p* > 0.05). In addition, the abundance of *Clostridium* cluster IV in the W3 group and *Clostridium* cluster XIVa in the W1 group was significantly higher than that in the C3 group (*p* < 0.05). It is indicated that *W. confusa* BSP201703 has an obvious dose-dependent bidirectional regulatory characteristic: the medium-dose W2 focuses on reconstructing the intestinal microbiota structure, improving overall community diversity and species abundance, and facilitating the recovery of microecological homeostasis, while the low-dose W1 and high-dose W3 achieve targeted enrichment of characteristic beneficial bacteria of Bacteroidetes and *Clostridium* clusters. Moreover, probiotic intervention did not significantly disturb the inherent abundances of *Lactobacillus* and Enterobacteriaceae, illustrating that its regulatory effect is mainly targeted at core homeostatic microbiota without blindly interfering with the conventional microbiota structure. In summary, different doses of *W. confusa* BSP201703 can effectively ameliorate intestinal microbiota dysbiosis caused by ETEC infection from two dimensions of macroscopic community restoration and microscopic targeted enrichment of beneficial bacteria, and the medium-dose W2 presents the optimal intervention effect.

## 4. Discussion

As a flagship species for global wildlife conservation and a national treasure of China, the giant panda (*Ailuropoda melanoleuca*) has benefited greatly from the optimization of captive breeding programs. Nevertheless, both captive and wild populations are still confronted with severe health threats. Intestinal bacterial infections, particularly those caused by enterotoxigenic *Escherichia coli* (ETEC), constitute a major pathogenic risk [8,11]. ETEC infection destroys the intestinal epithelial structure and impairs the integrity of the intestinal barrier, triggering clinical symptoms such as anorexia, weight loss, and even mortality in susceptible animals [12,32]. Critically, the intestinal microecological disturbance and mucosal damage induced by persistent ETEC colonization (rather than acute transient infection) accelerate the horizontal gene transfer among bacteria, enabling pathogenic ETEC to continuously accumulate virulence and drug-resistance genetic elements, and further evolve into multidrug-resistant extraintestinal pathogenic Escherichia coli (MDR-ExPEC). This greatly raises the risks of secondary infection, systemic spread, and cross-host transmission. Antibiotics remain the first-line therapy for bacterial enteritis. However, their overuse exacerbates gut microbiota dysbiosis and intensifies antibiotic selective pressure, further facilitating the prevalence and dissemination of high-risk strains such as MDR-ExPEC. These issues seriously threaten animal health and public health security and run counter to the core objectives of the global One Health initiative. In this context, probiotics with prominent intestinal barrier-protective functions are of vital importance. They can stabilize the intestinal mucosal structure, repair damaged barriers, block the colonization and invasion of ETEC at the source, inhibit the horizontal transmission of drug-resistance genes, and retard the evolutionary progression from ETEC to MDR-ExPEC. Such probiotics can alleviate infectious symptoms, restore intestinal microecological homeostasis, and reduce reliance on antibiotics, serving as a green and feasible strategy to control the transmission of drug-resistant pathogens and balance disease prevention with public health safety.

Probiotics have emerged as sustainable alternatives to antibiotics for controlling intestinal infectious diseases in animals, while effectively relieving the selective pressure that drives the emergence and dissemination of drug-resistant pathogens. They alleviate pathogen-induced intestinal injury without triggering antimicrobial resistance or gut dysbiosis associated with antibiotic application. Accumulating evidence supports the protective effects of probiotics against ETEC infection in various animal models and can further block horizontal transfer of resistance and virulence genes, thereby restraining the evolutionary progression of ETEC into MDR-ExPEC. For example, *Lactiplantibacillus plantarum* ZLP001 mitigates ETEC-induced downregulation of tight junction (TJ) proteins and excessive inflammatory responses [33]. It also inhibits pathogen adhesion and activates host defense pathways in pigs. Similarly, our previous study showed that *Lactiplantibacillus plantarum* G83 isolated from giant panda feces exerted strong protective effects in a mouse model of DSS-induced colitis [17]. These studies demonstrate that probiotics maintain intestinal homeostasis by regulating multiple physiological processes, curbing the enrichment and evolution of multidrug-resistant pathogenic strains, and thereby providing a solid theoretical basis for developing wildlife-derived probiotics to protect giant panda intestinal health and reduce the risk of MDR-ExPEC transmission.

A major limitation in research on giant panda intestinal health lies in the species’ protected status, which restricts direct in vivo experiments. Conventional animal models fail to recapitulate the giant panda intestinal microenvironment due to the high host specificity of gut microbiota. In this study, we established a giant panda fecal microbiota-associated (GPF) mouse model using antibiotic-pretreated mice. Our team has applied this technique to conduct systematic studies on gut microbiota diversity and abundance in various animals, including giant pandas [34], sheep [35], and rex rabbits [36]. Although PCR-DGGE confirmed that the model only partially recapitulated the gut microbiota characteristics of giant pandas, it effectively simulated their unique intestinal microenvironment and overcame the limitations of conventional models. Importantly, this model mimics the natural state of ETEC persistent colonization in giant pandas, providing a reliable platform for evaluating candidate probiotics against intestinal diseases in giant pandas.

The intestinal barrier comprises mechanical components (tight junction, TJ proteins), immune components (mucosal factors), and biological components (commensal microbiota), forming the first line of defense against enteric pathogens. Impaired barrier function represents an early and key event in the pathogenesis of ETEC-induced intestinal injury. In the present study, ETEC challenge induced obvious histopathological lesions, such as ileal mucosal hemorrhage in GPF mice. This finding is consistent with previous reports on ETEC pathogenicity [37] and confirms the model’s suitability for investigating intestinal barrier protection and pathogen intervention in a giant panda-relevant context. Notably, *W. confusa* BSP201703 alleviated ETEC-induced weight loss and reduced ileal mucosal damage, further validating our histopathological findings. Notably, ETEC-challenged groups showed higher ileal villus height and crypt depth compared to the blank control—a manifestation of compensatory epithelial hyperplasia during chronic ETEC infection, distinct from acute necrotic atrophy. *W. confusa* BSP201703 further optimized these morphological indicators, reflecting its role in repairing mucosal structural abnormalities induced by chronic colonization rather than simply inhibiting acute injury.

*Weissella* is a relatively understudied genus of lactic acid bacteria. In vitro studies have confirmed its favorable probiotic properties, including pathogen inhibition, acid and bile salt tolerance, and antioxidant activity [24,25]. However, relevant in vivo evidence remains limited. Recent reports indicate that *Weissella cibaria* WIKIM28 alleviates atopic dermatitis in mice by regulating immune responses, thereby demonstrating strong immunomodulatory potential [38]. Our preliminary in vitro tests showed that *W. confusa* BSP201703 produces higher exopolysaccharide, GABA, and bile salt hydrolase than *L. plantarum* G83 [39], suggesting superior intestinal protective potential. The present study is the first to verify the in vivo protective effects of *W. confusa* BSP201703. This strain effectively alleviated ETEC-induced intestinal injury and barrier dysfunction in GPF mice—filling the research gap regarding giant panda-derived *Weissella* in mitigating chronic ETEC infection. These findings support its potential application as a probiotic to improve intestinal health in giant panda.

Mechanistic analysis revealed that *W. confusa* BSP201703 upregulated mRNA expression of tight junction-related genes (*Tjp1*, *Ocln*, *Cldn-1*) in the jejunum and ileum, with significant differences detected in the ileum (*p* < 0.05). These changes contributed to enhanced intestinal mechanical barrier integrity. The probiotic treatment also elevated mucosal secretory immunoglobulin A (SIgA) levels and reduced serum D-lactate concentrations, a key biomarker of intestinal permeability. Notably, D-lactate levels in the W1 group were significantly lower than those in group C2 (*p* = 0.0114), indicating that this probiotic alleviates intestinal mucosal injury and restores microbial balance disrupted by antibiotic exposure and ETEC challenge. Furthermore, qPCR analysis revealed no marked alterations in the abundances of dominant intestinal taxa, including *Lactobacillus* and Enterobacteriaceae. These findings indicate that *W. confusa* BSP201703 alleviates ETEC infection primarily by enhancing intestinal mechanical and immune barrier functions, rather than inducing extensive restructuring of the gut microbiota. This distinct mechanism differentiates the strain from conventional probiotics, reflecting the unique functional specificity of this wildlife-derived isolate.

Notably, low and medium doses of *W. confusa* BSP201703 conferred stronger protective effects than the high dose, with the highest microbial richness detected in group W2. This does not represent a typical biphasic dose–response pattern (which involves opposite effects at low vs. high doses) but rather an optimal dosage window effect—intestinal protection does not increase monotonically with dosage but peaks at the medium concentrations. The underlying mechanism may involve excessive immune activation, mild gut ecological disturbance, or intensified metabolic competition at high dosing—factors that can counteract beneficial effects and weaken intestinal protection. These clear dose-dependent differences emphasize that rational dosage optimization is critical to maximizing the probiotic’s protective efficacy against ETEC-induced intestinal injury.

Several limitations of this study should be acknowledged. Firstly, the GPF mouse model only partially recapitulates the native gut microbiota of giant pandas, which may restrict the direct extrapolation of these findings to captive or wild giant pandas. Secondly, no prominent microbial shifts were detected by qPCR, indicating that further high- throughput sequencing is required. In addition, combined approaches such as labeling the strain and applying small-animal three-dimensional imaging systems will help clarify its protective mechanism against intestinal barrier injury in greater detail. Thirdly, although the medium dose of *W. confusa* BSP201703 exhibited superior efficacy compared with the high dose, this optimal dosage still needs further validation in larger animal models and targeted trials using captive giant pandas. Fourthly, the intestinal barrier-protective properties of this strain could be further exploited to prevent and control infections caused by multidrug-resistant *Escherichia coli* in giant pandas, expanding its application value under the One Health framework.

## 5. Conclusions

Giant panda-derived *Weissella confusa* BSP201703 effectively alleviates intestinal mucosal damage caused by persistent ETEC infection, with its protective effects mainly relying on enhancing the intestinal mechanical barrier and mucosal immune function, rather than large-scale regulation of the gut microbiota. The GPF mouse model established in this study, which simulates the unique intestinal microenvironment of giant pandas, provides a reliable platform for exploring probiotic intervention strategies against ETEC-induced intestinal damage. The strain does not show a typical biphasic dose–response relationship, but presents an optimal dosage window effect—low and medium doses of probiotics are more conducive to repairing chronic intestinal damage. This study enriches the functional research of wildlife-derived probiotics and provides practical support for the application of native probiotics in protecting giant panda health. Meanwhile, it promotes the implementation of the One Health concept in wildlife disease control and conservation, laying a solid foundation for the sustainable development of giant panda conservation and intestinal health management.

## Figures and Tables

**Table 1:** Primer sequences for quantitative PCR of tight junction protein genes.

Genes	Sequence (5′-3′)	Tm (°C)
ZO-1	F-GATCCCTGTAAGTCACCCAGA R-CTCCCTGCTTGCACTCCTATC	60
Occludin	F-TTGAAAGTCCACCTCCTTACAGA R-CCGGATAAAAAGAGTACGCTGG	60
Claudin-1	F-GGGGACAACATCGTGACCG R-AGGAGTCGAAGACTTTGCACT	60

**Table 2:** Primer sequences for quantitative PCR of bacteria.

Bacteria Species	Sequence (5′-3′)	Length (bp)	Tm (°C)
Firmicutes	F-GCAGYATGTGGTTTAATTCGAAGCA R-AGCTGACGACAACCATGCAC	126	58
Bacteroidetes	F-GGARCATGTGGTTTAATTCGATGAT R-AGCTGACGACAACCATGCAG	126	58
*Lactobacillus*	F-AGCAGTAGGGAATCTTCCA R-CACCGCTACACATGGAG	341	55
Enterobacteriaceae	F-CCCTTATTGTTAGTTGCCATCATT R-ACTCGTTGTACTTCCCATTGT	144	60
*Clostridium* group IV	F-GCACAAGCAGTGGAGT R-CTTCCTCCGTTTTGTCAA	130	60
*Clostridium* group XIVa	F-AAATGACGGTACCTGACTAA R-CTTTGAGTTTCATTCTTGCGAA	440	60

## Data Availability

The original contributions presented in this study are included in the article. Further inquiries can be directed to the corresponding authors.

## Abbreviations

The following abbreviations are used in this manuscript:

GPF	Giant panda fecal microbiota-associated
ABX	Antibiotic-treated
TJ	Tight junction
ZO-1	Zonula Occludens-1
qPCR	Quantitative real-time PCR
PCR-DGGE	Polymerase chain reaction-denaturing gradient gel electrophoresis
ETEC	Enterotoxigenic *Escherichia coli*
EHEC	Enterohemorrhagic *Escherichia coli*
ELISA	Enzyme-linked immunosorbent assay
SIgA	secretory immunoglobulin A
